# Measurements of equine foot parameters show limited agreement between radiographs and low‐field magnetic resonance imaging

**DOI:** 10.1111/evj.14536

**Published:** 2025-06-26

**Authors:** Constance Bowkett‐Pritchard, David M. Bolt, Yu‐Mei Chang, Dagmar Berner

**Affiliations:** ^1^ Equine Referral Hospital, Department of Clinical Science and Services Royal Veterinary College London UK; ^2^ The Philip Leverhulme Equine Hospital University of Liverpool Neston; ^3^ Department of Comparative Biomedical Sciences Royal Veterinary College London UK

**Keywords:** hoof, horse, MRI, radiography

## Abstract

**Background:**

Equine foot radiographs are commonly obtained to measure anatomical conformation parameters. Comparison of measurements between radiographs and low‐field magnetic resonance imaging (MRI) has not been extensively explored.

**Objectives:**

To compare foot parameter measurements between radiographs and low‐field MRI, and assess the effect of hoof wall markers on visualising the hoof capsule (during MRI) and facilitating measurements.

**Study Design:**

Comparative cadaveric analytical study.

**Methods:**

Radiography and MRI of nine equine cadaver front feet were performed with and without hoof wall markers, which were lead strips for radiography and a water‐soaked hoof bandage for MRI. Intra‐observer reliability and inter‐modality agreement were calculated using intra‐class correlation coefficients (ICC) with 95% confidence intervals (CI) and *p* < 0.05.

**Results:**

Intra‐observer repeatability was generally good, apart from distal dermal frontal measurements. There was limited agreement between radiographic and MRI measurements. Results are presented as RAD indicating those obtained with radiography and T1, T2* or STIR indicating those obtained with the relevant MRI sequence; m is added if a marker was used. Founder distance only showed good agreement for radiographic and T1 measurements with markers; ICC 0.78 (CI 0.33–0.95 *p* = 0.004). Inter‐modality comparisons for distal phalanx rotation were limited by intraobserver repeatability. Good agreement was noted for sole thickness and epidermal sole thickness measurements with markers; sole thickness (RADm vs. T1m ICC 0.81 [CI −0.04‐0.96], *p* < 0.001; RADm vs. T2*m ICC 0.86 [CI 0.51–0.97], *p* < 0.001; RADm vs. STIRm ICC 0.91 [CI 0.66–0.98], *p* < 0.001) and epidermal sole thickness (RADm vs. T1m ICC 0.88 [CI 0.55–0.97], *p* < 0.001; RADm vs. T2*m ICC 0.83 [CI 0.41–0.96], *p* = 0.002; RADm vs. STIRm ICC 0.80 [CI 0.31–0.95], *p* = 0.004). Radiographic measurements with and without markers often had good to excellent agreement; for some parameters, hoof wall markers were associated with reduced intra‐observer repeatability. The water‐soaked hoof bandage aided MRI hoof capsule visualisation; limitations included reduced repeatability and unattainable distal measurements.

**Main Limitations:**

Small sample size.

**Conclusions:**

The limited agreement between radiographic and MRI measurements suggests these modalities are not interchangeable in equine foot assessment. Hoof wall markers do not benefit foot measurements.

## 1 INTRODUCTION

A cornerstone in imaging assessment of equine feet involves measuring the relationship between the distal phalanx (P3) and the hoof capsule.^1^, ^2^, ^3^, ^4^, ^5^, ^6^ Magnetic resonance imaging (MRI) facilitates the diagnosis of multiple foot soft tissue pathologies,^7^ provides detail regarding equine hoof disorders, including laminitis,^1^, ^2^, ^7^ and provides the opportunity to assess the relationship between the hoof capsule and P3. The comparability between radiographic and MRI measurements of equine foot parameters is unclear, as previous studies have yielded variable results.^1^, ^2^, ^4^, ^8^

One cadaver study demonstrated good agreement between radiography and MRI, particularly for dorsal hoof wall measurements.^4^ Another study found MRI to be more sensitive and specific for identification of laminitis‐related parameters, but results varied between radiographs and MRI.^1^ A further study into laminitis‐related parameters found no difference for distal phalanx rotation angle (P3rotang), but radiographic measurements of founder distance and dorsal hoof wall thickness (DHWT) were significantly greater than MRI.^2^ These studies used a magnetic field strength of 1.5–4.7 T. A low‐field (0.27 T) study found significant differences between select radiographic and MRI foot measurements.^8^ Further investigation of the comparability of radiographic and low‐field MRI foot measurements is warranted, given that low‐field MRI systems are commonly used in equine clinical practice.

Dorsal hoof wall markers, such as wire, barium paste, metallic or lead strips, are commonly used to highlight the hoof capsule on radiographs.^6^, ^9^ Normally, the hoof wall is not visible on MRI due to decreasing water content from deep to superficial areas,^10^ causing low MRI signal.^1^ This has previously been overcome by applying lard,^4^ plasticine,^2^ or a wet gauze foot bandage.^11^ Studies investigating the effect of hoof wall markers on radiographic and MRI foot measurements are lacking; one study found no benefit of a dorsal hoof wall marker on radiographs.^9^

This study's primary aim was to assess the agreement between an extensive range of hoof measurements obtained from radiographs and low‐field MRI. Secondary aims were to validate a wet hoof bandage to visualise the hoof wall outline during low‐field MRI and to investigate the effect of hoof wall markers (lead strips [radiographs] and a water‐soaked hoof bandage [MRI]) on these measurements. These markers are readily available in clinical practice. We hypothesised that (i) the water‐soaked hoof bandage would permit visualisation of the hoof capsule for MRI measurements, (ii) MRI and radiographic foot measurements would show limited agreement, regardless of hoof wall marker use, and (iii) hoof wall marker (either lead strips or the water‐soaked hoof bandage) would not affect foot measurement values.

## 2 MATERIALS AND METHODS

An analytical study was performed using a convenience sample of nine equine distal cadaver forelimbs (five right and four left) obtained from an abattoir. Horses were of unknown signalment and euthanised for unknown reasons. Only limbs with feet considered normal on gross inspection (no obvious hoof wall defects or deviations) and imaging examination (radiographs and MRI, as described below) were included. This was determined by an ECVDI board‐certified radiologist (Dagmar Berner).

### 2.1 Radiography

**FIGURE 1:**
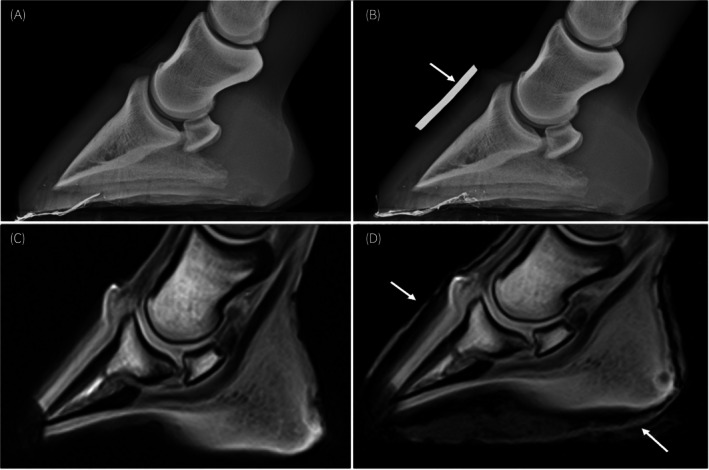
(A, B) Lateromedial radiographs (64 kV, 1.7 mAs) and (C, D) T1W gradient recall echo sagittal sequences of a cadaver foot. In (B), a radiopaque hoof wall marker (arrow) is placed on the dorsal hoof wall adjacent to the coronary band. In (D), the arrows indicate the water‐soaked hoof bandage placed around the foot. The loss of the dorsodistal toe region in images (C) and (D) could be due to this structure being at the edge of the field of view, the need for a thicker bandage, or susceptibility artefact from radiographically invisible metallic clench fragments.

Prior to imaging, the feet were cleaned and barium sulphate (97%, Source Chemicals) paste was placed in a linear fashion from the frog apex to the toe. Lateromedial and dorsopalmar radiographic projections were obtained with the limbs balanced in an upright position on a wooden foot block, centred on the coronary band. The generator to plate distance was 100 cm. Images were obtained using a digital radiography system; a Powerlight 90 generator (veterinary x‐rays) (settings: 64 kV, 1.7 mAs) and Fujifilm detector (FUJIFILIM DR‐ID 613SE [24 × 30] flat panel sensor [FUJIFLIM UK Ltd]). Projections were repeated with linear 4 mm thick radiodense lead markers placed by the same operator (Constance Bowkett‐Pritchard). For each lateromedial projection, the marker was placed on the sagittal midline along the dorsal hoof wall, using adhesive tape, with the proximal aspect touching the coronary band (Figure 1A,B). For the dorsopalmar projections, two markers were placed in a proximodistal orientation, one medially and one laterally at the widest part of the hoof wall, touching the coronary band proximally. The radiographic markers and any metal clenches seen radiographically were then removed prior to MRI examination.

### 2.2 Magnetic resonance imaging

The foot was balanced on a flat surface in an upright position and scanned using a 0.27 T low‐field MRI system (EQ2, Hallmarq) and a dedicated hoof coil; T1 weighted 3D GRE (gradient recall echo), T2* weighted 3D GRE and short tau inversion recovery (STIR) fast spin echo (FSE) sequences were obtained in sagittal and frontal planes (Table 1), reflecting common clinical protocols. Depending on the STIR test images, either a STIR FSE (−) or STIR FSE sequence was used. Piloting was standardised, with centring of the cross hairs on the palmarodistal aspect of the middle phalanx. For frontal scans, piloting was parallel to the deep digital flexor tendon. Images were acquired before and after wrapping the foot in a single layer of cotton wool bandage (Soffban, BSN Medical Ltd) (Figure 1C,D). The frog sulci were also packed with the same cotton wool. The foot was then covered by an elasticated adhesive dressing (Vetwrap, 3 M) and submerged in water until the bandage was soaked.

**TABLE 1:** Low‐field MRI sequence parameters used to image cadaver limbs.

Sequence	TE in ms	TR in ms	TI in ms	Flip angle	ST in mm	IG in mm	Plane
T1W 3D GRE	7	24	NA	45	3	0	Sagittal
T1W 3D GRE HR	8	24	NA	45	1.5	0	Frontal
T2*W 3D GRE	13	34	NA	25	3	0	Sagittal and Frontal
STIR FSE 2D (−)^a^	27	2742	70	90	5	1	Sagittal and frontal
STIR FSE 2D^a^	27	3042	95	90	5	1	Sagittal and frontal

Abbreviations: FSE, fast spin echo; GRE, gradient recall echo; HR, high resolution; IG, interslice gap; ST, slice thickness; STIR, short tau inversion recovery; TE, echo time; TI, inversion time; TR, repetition time; W, weighted.

^a^ Depending on the STIR test images, either a STIR FSE (−) or STIR FSE sequence was used.

### 2.3 Measurements

**FIGURE 2:**
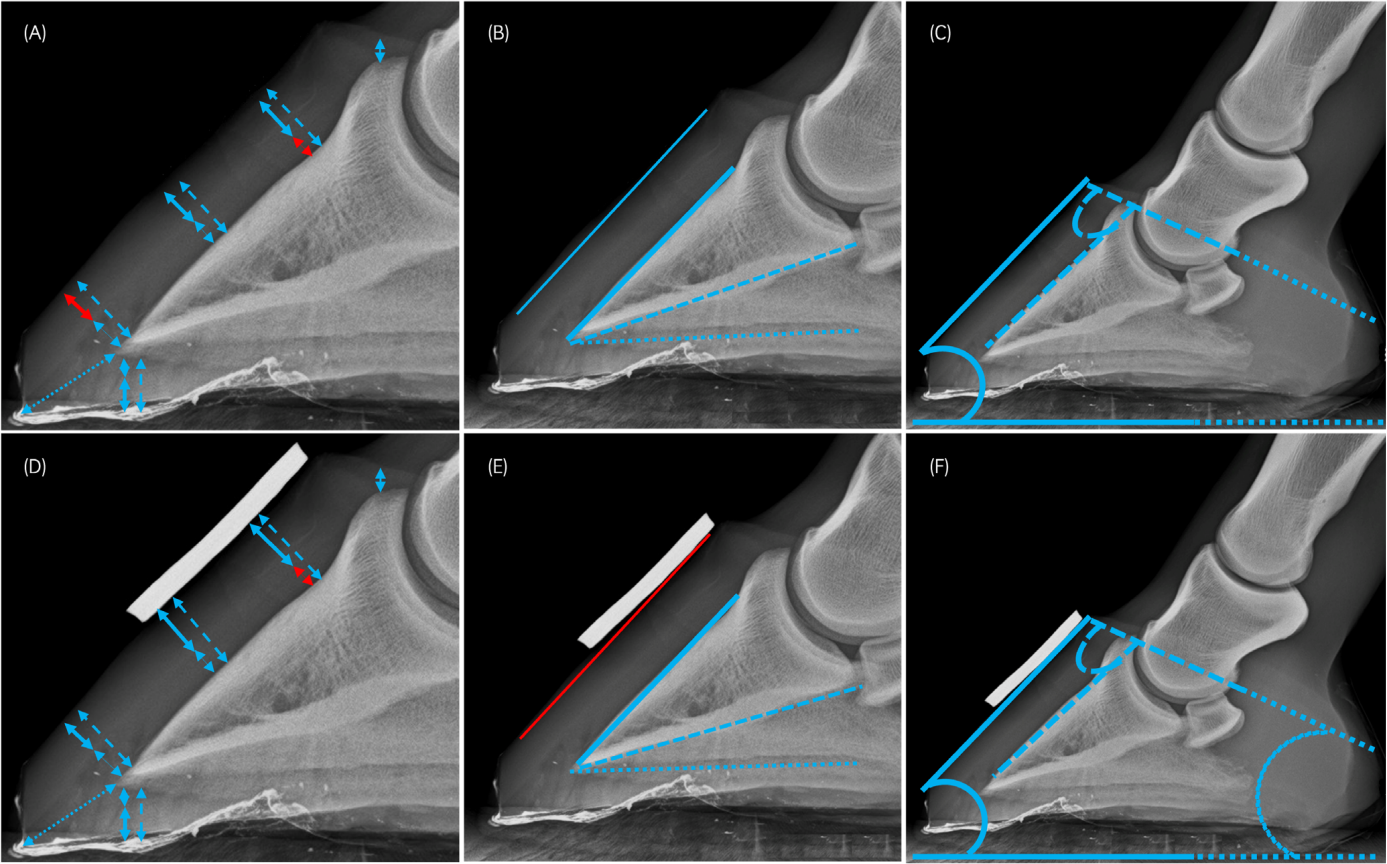
Lateromedial radiographs of a cadaver foot (64 kV, 1.7 mAs) without (A–C) and with (D–F) markers, demonstrating measurements performed. Measurements in blue indicate intra‐observer repeatability with an intraclass correlation (ICC) >0.7; measurements in red indicate intra‐observer repeatability with ICC <0.7. A glossary of measurement abbreviations is provided in Table 2. (A, D) Dorsal and solar measurements; hoof wall thickness (DHWT) and sole thickness (dashed double arrow), epidermal thickness (DEWT and EST) (bold double arrow) and dermal thickness (DDWT and DST) (thin double arrow). The double arrow between the distal tip of the distal phalanx and the toe indicates toe length. The double arrow between the distal phalanx extensor process and the coronary band indicates founder distance. (B, E) Dorsal P3 (DP3ang—solid blue line), distal P3 (DiP3ang—dashed line) and solar (dotted line) angles relative to the weight bearing surface of the foot. P3 rotation is shown by the solid line overlying the dorsal hoof capsule relative to dorsal P3. (C, F) Hoof capsule (HCang—solid lines), coronet to P3 (CP3ang—dashed line) and coronet (dotted line) angles.

**FIGURE 3:**
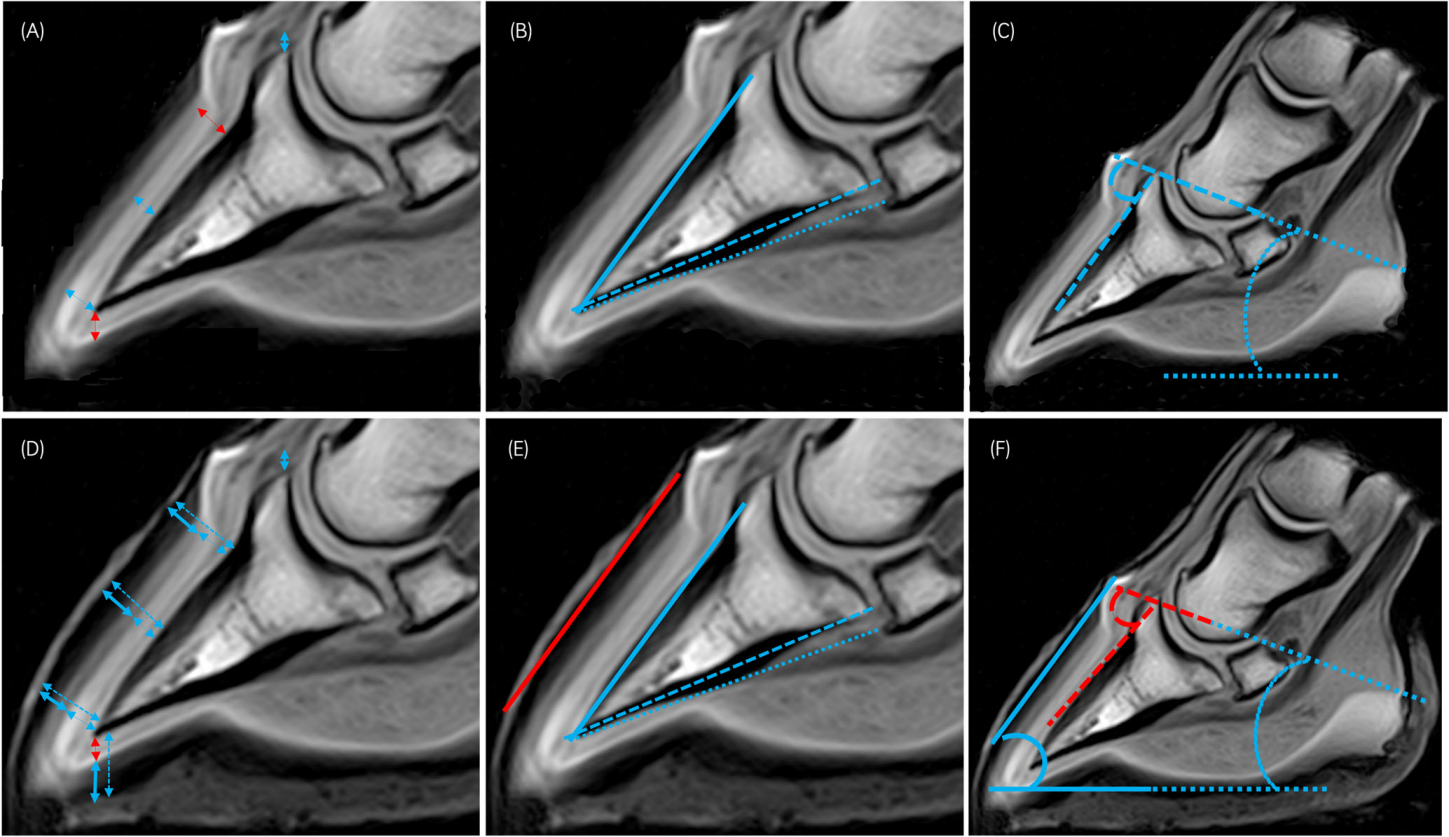
T1 weighted gradient recall echo sagittal images without (A–C) and with (D–F) a water‐soaked hoof bandage, demonstrating measurements performed. Measurements in blue indicate intra‐observer repeatability with an intraclass correlation (ICC) >0.7; measurements in red indicate intra‐observer repeatability with ICC <0.7. A glossary of measurement abbreviations is provided in Table 2. (A, D) Dorsal and solar measurements; hoof wall thickness (DHWT and sole thickness) (dashed double arrow), epidermal thickness (DEWT and EST) (bold double arrow) and dermal thickness (DDWT and DST) (thin double arrow). The double arrow between the distal phalanx extensor process and the coronary band indicates founder distance. Toe length measurements are not possible because the distal hoof capsule is not visible. (B, E) Dorsal P3 (DP3ang—solid line), distal P3 (DiP3ang—dashed line) and solar (Sang—dotted line) angles. P3 rotation (P3rotang) is shown by the solid lines overlying the dorsal hoof capsule relative to dorsal P3. (C, F) Hoof capsule (HCang—solid lines), coronet to P3 (CP3ang—dashed line) and coronet (Cang—dotted line) angles.

**FIGURE 4:**
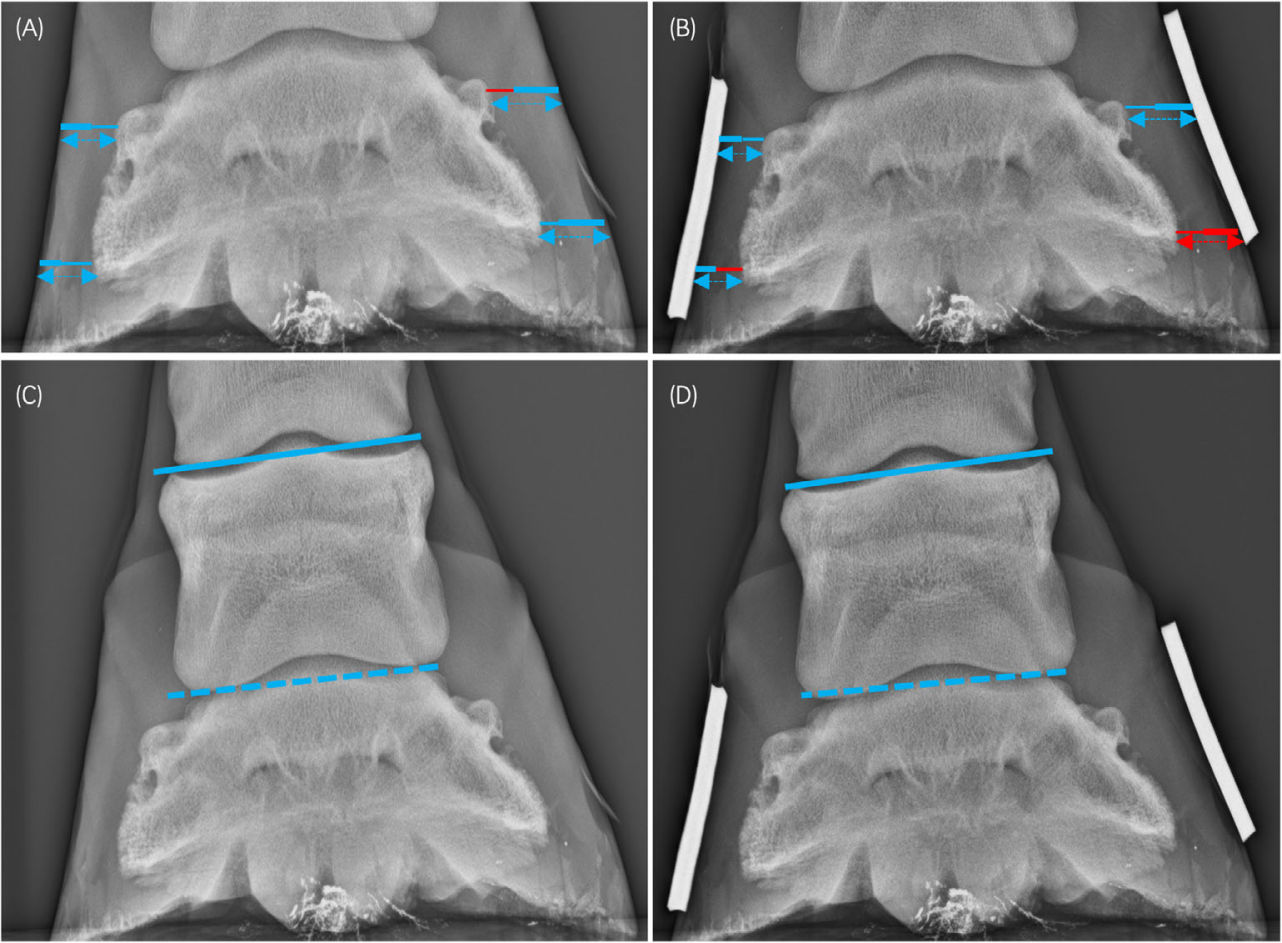
Dorsopalmar radiographs (64 kV, 1.7 mAs) without (A, C) and with (B, D) hoof wall markers, demonstrating measurements performed. Lateral is to the right of the images. Measurements in blue indicate intra‐observer repeatability with an intraclass correlation (ICC) >0.7; measurements in red indicate intra‐observer repeatability with ICC <0.7. A glossary of measurement abbreviations is provided in Table 2. Proximal (Pr) and distal (Di) medial (M) (A, D) and lateral (L) (B, E) hoof wall thickness (HWT‐dashed double arrow), epidermal thickness (EWT‐bold line) and dermal thickness (DWT‐thin line). The solid and dashed lines (C, D) indicate the proximal and distal middle phalanx rotation angle, PrP2ang and DiP2ang, respectively.

**FIGURE 5:**
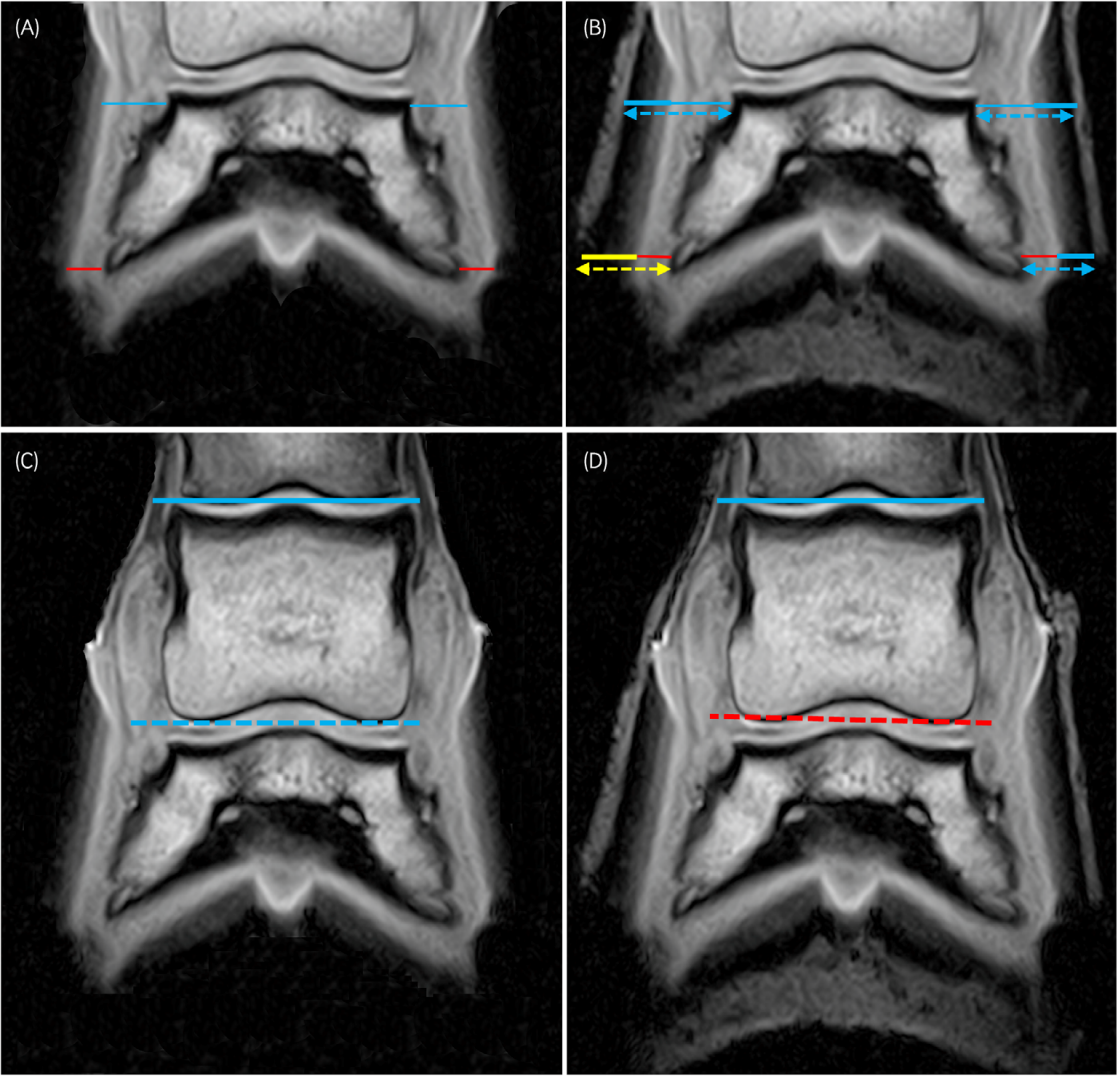
T1 weighted gradient recall echo frontal images without (A and C) and with (B and D) a water‐soaked hoof bandage, demonstrating measurements performed. Measurements in blue indicate intra‐observer repeatability with an intraclass correlation (ICC) >0.7; measurements in red indicate intra‐observer repeatability with ICC <0.7. Measurements in yellow indicate excessive unattainable values. A glossary of measurement abbreviations is provided in Table 2. Proximal (Pr) and distal (Di) medial (M) and lateral (L) hoof wall thickness (HWT‐dashed double arrow), epidermal thickness (EWT‐bold line) and dermal thickness (DWT‐thin line). The solid and dashed lines (C and D) indicate the proximal and distal middle phalanx rotation angle, PrP2ang and DiP2ang, respectively.

Images were anonymised and a range of distance and angle measurements was obtained by a single blinded observer (Constance Bowkett‐Pritchard; a veterinary surgeon of 4 years' experience) on three occasions, at least 2 weeks apart, using digital imaging software (Horos). The scheme and abbreviations used for measurements is presented in Table 2. Use of ‘m’ indicates a measurement acquired with a marker; ‘RAD’ indicates a radiographic measurement and ‘T1’, ‘T2*’ or ‘STIR’ indicate an MRI measurement from the respective sequence. On lateromedial radiographic and sagittal MR images, these included P3 length (P3L), dorsal (DHWT), epidermal (DEWT) and dermal wall thickness (DDWT) (proximal, middle and distal), founder distance, P3 rotation angle (P3rotang), solar angle, sole thickness, epidermal (EST) and dermal sole thickness (DST), toe length, hoof capsule angle (HCang), dorsal P3 angle (DP3ang), distal P3 angle (DiP3ang), coronet angle and coronet to P3 angle (CP3ang) (Figures 2 and 3). Measurements obtained using dorsopalmar radiographs and frontal MR images included proximal and distal, medial (M) and lateral (L) hoof (HWT), epidermal (EWT) and dermal (DWT) wall thickness and proximal and distal P2 to solar angle (PrP2ang and DiP2ang, respectively) (Figures 4 and 5). MRI measurements were obtained at the midpoint slice of the frontal and sagittal scans. For the frontal measurements, the phalangeal column and P3 vascular channels were used as landmarks to ensure consistency. Table S1 contains the measurement protocol.

**TABLE 2:** Glossary of measurement abbreviations.

Abbreviation	Term
CP3ang	Coronet to distal phalanx angle
DDWT	Dorsal dermal wall thickness
DHWT	Dorsal hoof wall thickness
LDWT	Lateral dermal wall thickness
LEWT	Lateral epidermal wall thickness
LHWT	Lateral hoof wall thickness
MDWT	Medial dermal wall thickness
MEWT	Medial epidermal wall thickness
MHWT	Medial hoof wall thickness
DiP2ang	Distal middle phalanx to solar angle
DiP3ang	Distal axis of distal phalanx angle
DP3ang	Dorsal distal phalanx angle
DST	Dermal solar thickness
EST	Epidermal solar thickness
HCang	Hoof capsule angle
m	Added to indicate a marker was used
P2	Middle phalanx
P3	Distal phalanx
P3L	Distal phalanx length
P3rotang	Distal phalanx rotation angle
PrP2ang	Proximal middle phalanx to solar angle

### 2.4 Data analysis

Data were collated on a spreadsheet (Microsoft Excel). Statistical analysis was performed using a commercially available software (SPSS Statistical Package, Version 29, SPSS Inc.). To investigate intra‐observer repeatability, agreement between the three separate measurements for each parameter was assessed by calculating the intraclass correlation coefficient (ICC; two‐way mixed, absolute agreement model) with a 95% confidence interval. Only parameters with acceptable repeatability (defined as ICC >0.7^12^) were used for further analysis. Measurements for which some values were unattainable (due to indistinct anatomical landmarks) were also recorded. For any parameter, if five or more measurements (out of the nine limbs) were unattainable, this was defined as ‘excessive unattainable values’ and the parameter was excluded from analysis.

For remaining parameters with acceptable repeatability and attainability, a mean value of the three measurements was calculated. All length and thickness measurements were divided by the distal phalanx length (P3L), creating a ratio to account for magnification.^13^ Angle measurements remained as raw values.

To investigate inter‐modality agreement, ICC was calculated as above for each remaining parameter. Comparisons were made between radiographic and MRI measurements, with and without hoof wall markers. Statistical significance was set at *p* < 0.05. Classification of inter‐modality ICCs was: poor <0.5, moderate 0.5–0.75, good 0.75–0.90 and excellent >0.9.^12^

## 3 RESULTS

### 3.1 Intraobserver repeatability

Detailed descriptive and intraobserver reliability data are displayed in Tables S2–S4 and Figures 2, 3, 4, 5.

Intraobserver repeatability did not always meet the ICC threshold of 0.7 for laminitis measurements. For founder distance, measurement repeatability was above the threshold for radiographic and T1 measurements (with and without markers) and T2* measurements without markers. Only plain radiographic and T2* P3rotang measurements with markers exceeded the threshold.

The ICC exceeded 0.75 for most measurements involving the dorsal hoof wall, all solar wall thickness and solar epidermal thickness measurements (radiographs and MRI) as well as solar dermal thickness (radiographs only) and toe length (radiographs and STIR MRI only).

Intraobserver repeatability was variable for frontal hoof wall measurements. Distal and dermal measurements tended to have poorer repeatability compared to proximal epidermal and wall thickness measurements.

Intraclass correlations for all radiographic angle measurements, with and without markers, exceeded 0.7 but were variable for MRI measurements; T2* and STIR measurements were often below the threshold. Intraclass correlations for frontal MRI measurements of PrP2ang and DiP2ang often exceeded the threshold without markers but were below 0.5 with markers.

### 3.2 Hypothesis 1: Use of the water‐soaked hoof bandage

MRI measurements involving the hoof capsule were not possible without the water‐soaked hoof bandage. Despite its use, some T1 and T2* measurements remained unattainable; these involved distal frontal and toe length measurements. No angle measurement was unattainable.

### 3.3 Hypothesis 2: Agreement between MRI and radiographic foot measurements

Detailed inter‐modality comparison data are displayed in Tables 3 and 4.

**TABLE 3:** Intermodality comparison for each measurement ratio (comparisons between measurements with poor repeatability and or excessive unattainable values are excluded) using an intra‐class correlation (ICC) with 95% confidence intervals (CI).

Measurement	Comparison	Intra‐class correlation	ICC rating	Lower confidence interval	Upper confidence interval	*p*
Dorsal hoof wall measurements						
PrDHWT	*RAD* versus *RADm*	*0.88*	*Good*	*0.58*	*0.97*	*<0.001*
	RADm versus T1m	0.70	Moderate	−0.04	0.93	0.001
	RADm versus T2*m	0.71	Moderate	−0.03	0.93	0.001
	*RADm* versus *STIRm*	*0.86*	*Good*	*0.37*	*0.97*	*<0.001*
MiDHWT	RAD versus RADm	0.68	Moderate	0.14	0.92	0.01
	RADm versus T1m	0.58	Moderate	0.0	0.88	0.02
	RADm versus T2*m	0.51	Moderate	−0.15	0.86	0.07
	RADm versus STIRm	0.74	Moderate	0.16	0.93	0.01
DiDHWT	*RAD* versus *RADm*	*0.97*	*Excellent*	*0.88*	*0.99*	*<0.001*
	*RADm* versus *T1m*	*0.91*	*Excellent*	*0.64*	*0.98*	*<0.001*
	*RADm* versus T2*m	*0.80*	*Good*	*0.17*	*0.96*	*<0.001*
	RADm versus STIRm	0.61	Moderate	−0.10	0.91	0.002
PrDDWT	T2*versus T2*m	0.69	Moderate	0.14	0.92	0.01
MiDDWT	*RAD* versus *RADm*	*0.86*	*Good*	*0.51*	*0.97*	*<0.001*
	RAD versus T1	0.65	Moderate	−0.01	0.91	0.02
	RAD versus T2*	0.56	Moderate	−0.09	0.88	0.05
	RAD versus STIR	0.46	Poor	−0.20	0.84	0.09
	*RADm* versus *T1m*	*0.83*	*Good*	*0.14*	*0.97*	*<0.001*
	*RADm* versus T2*m	*0.88*	*Good*	*0.54*	*0.97*	*<0.001*
	*RADm* versus *STIRm*	*0.94*	*Excellent*	*0.75*	*0.99*	*<0.001*
	T1 versus T1m	0.71	Moderate	0.19	0.92	0.008
	T2* versus T2*m	*0.75*	*Good*	*0.26*	*0.94*	*0.006*
	STIR versus STIRm	0.68	Moderate	0.10	0.92	0.02
DiDDWT	*RAD* versus *RADm*	*0.84*	*Good*	*0.48*	*0.96*	*<0.001*
	RAD versus T1	0.70	Moderate	0.10	0.93	0.02
	RAD versus T2*	0.52	Moderate	−0.10	0.87	0.02
	RAD versus STIR	0.42	Poor	−0.08	0.83	0.003
	RADm versus T1m	0.69	Moderate	0.13	0.92	0.006
	*RADm* versus T2*m	*0.81*	*Good*	*0.38*	*0.95*	*0.003*
	RADm versus STIRm	0.48	Poor	−0.12	0.85	0.02
	T1 versus T1m	0.69	Moderate	0.16	0.93	0.01
	T2*versus T2*m	0.60	Moderate	0.02	0.90	0.03
	STIR versus STIRm	0.71	Moderate	0.10	0.93	0.02
PrDEWT	*RAD* versus *RADm*	*0.87*	*Good*	*0.53*	*0.97*	*<0.001*
	*RADm* versus *T1m*	*0.81*	*Good*	*0.28*	*0.96*	*<0.001*
	RADm versus T2*m	0.62	Moderate	0.01	0.90	0.01
	*RADm* versus *STIRm*	*0.81*	*Good*	*0.35*	*0.95*	*0.003*
MiDEWT	RAD versus RADm	0.42	Poor	−0.20	0.82	0.10
	RADm versus T1m	0.18	Poor	−0.48	0.73	0.31
	RADm versus T2*m	0.05	Poor	−0.55	0.65	0.45
	RADm versus STIRm	0.34	Poor	−0.41	0.81	0.18
DiDEWT	*RADm* versus *T1m*	*0.91*	*Excellent*	*0.59*	*0.98*	*<0.001*
	*RADm* versus T2*m	*0.81*	*Good*	*0.31*	*0.96*	*<0.001*
	RADm versus STIRm	0.73	Moderate	0.22	0.93	0.008
Frontal measurements						
DiLDWT	RAD versus STIR	0.07	Poor	−0.13	0.47	0.3
DiMDWT	RAD versus STIR	0.00	Poor	−0.20	0.42	0.5
DiMEWT	*RAD* versus *RADm*	*0.77*	*Good*	*0.32*	*0.94*	*0.004*
	RADm versus STIRm	0.46	Poor	−0.12	0.84	0.06
DiMHWT	*RAD* versus *RADm*	*0.94*	*Excellent*	*0.79*	*0.99*	*<0.001*
	RADm versus T1m	0.25	Poor	−0.18	0.76	0.1
	RADm versus STIRm	0.30	Poor	−0.17	0.75	0.1
PrLDWT	RADm versus T1m	0.01	Poor	−0.04	0.18	0.4
	T1 versus T1m	0.55	Moderate	−0.17	0.88	0.06
PrLEWT	RAD versus RADm	0.34	Poor	−0.18	0.78	0.1
	RADm versus T1m	0.04	Poor	−0.14	0.44	0.4
	RADm versus STIRm	0.22	Poor	−0.24	0.70	0.2
PrLHWT	RAD versus RADm	0.75	Moderate	0.22	0.94	0.003
	RADm versus T1m	0.03	Poor	−0.03	0.23	0.3
	RADm versus T2*m	0.02	Poor	−0.03	0.19	0.3
PrMDWT	*RAD* versus *RADm*	*0.91*	*Excellent*	*0.64*	*0.98*	*<0.001*
	RAD versus T1	−0.02	Poor	−0.04	0.10	0.9
	RAD versus T2*	−0.02	Poor	−0.03	0.08	0.9
	RAD versus STIR	−0.01	Poor	−0.05	0.13	0.7
	RADm versus T1m	0.05	Poor	−0.02	0.30	0.05
	RADm versus T2*m	0.02	Poor	−0.01	0.16	0.1
	T1 versus T1m	0.25	Poor	−0.56	0.78	0.3
	T2* versus T2*m	0.68	Moderate	0.05	0.92	0.02
PrMEWT	RAD versus RADm	0.72	Moderate	0.19	0.93	0.01
	RADm versus T1m	0.16	Poor	−0.16	0.62	0.2
	RADm versus STIRm	0.46	Poor	−0.16	0.84	0.08
PrMHWT	*RAD* versus *RADm*	*0.81*	*Good*	*0.40*	*0.95*	*0.002*
	RADm versus T1m	0.05	Poor	−0.02	0.29	0.05
	RADm versus T2*m	0.05	Poor	−0.02	0.28	0.05
	RADm versus STIRm	0.04	Poor	−0.04	0.28	0.2
Solar measurements						
Sole thickness	*RAD* versus *RADm*	*0.98*	*Excellent*	*0.92*	*>0.99*	*<0.001*
	*RADm* versus *T1m*	*0.81*	*Good*	*−0.04*	*0.96*	*<0.001*
	*RADm* versus T2*m	*0.86*	*Good*	*0.51*	*0.97*	*<0.001*
	*RADm* versus *STIRm*	*0.91*	*Excellent*	*0.66*	*0.98*	*<0.001*
EST	*RAD* versus *RADm*	*0.93*	*Excellent*	*0.71*	*0.98*	*<0.001*
	*RADm* versus *T1m*	*0.88*	*Good*	*0.55*	*0.97*	*<0.001*
	*RADm* versus T2*m	*0.83*	*Good*	*0.41*	*0.96*	*0.002*
	*RADm* versus *STIRm*	*0.80*	*Good*	*0.31*	*0.95*	*0.004*
DST	*RAD* versus *RADm*	*0.83*	*Good*	*0.43*	*0.96*	*0.002*
	RAD versus STIR	0.17	Poor	−0.54	0.73	0.3
Toe length	*RAD* versus *RADm*	*0.93*	*Excellent*	*0.72*	*0.98*	*<0.001*
	RADm versus T1m	0.71	Moderate	0.08	0.94	0.01
	RADm versus T2*m	0.66	Moderate	−0.08	0.93	0.003
	RAD versus STIRm	0.73	Moderate	0.13	0.93	0.0002
Laminitis measurements						
Founder distance	*RAD* versus *RADm*	*0.76*	*Good*	*0.29*	*0.94*	*0.004*
	RAD versus T1	0.48	Poor	−0.12	0.85	0.01
	RAD versus T2*	0.59	Moderate	−0.10	0.90	0.004
	*RADm* versus *T1m*	*0.78*	*Good*	*0.33*	*0.95*	*0.004*
	T1 versus T1m	0.54	Moderate	−0.13	0.87	0.06

*Note*: Lines in italics highlight measurements with good (ICC 0.75–0.90) or excellent (ICC >0.9) agreement; we draw the reader's attention to the comparatively small number of measurements that met these criteria compared to measurements with moderate (ICC 0.5–0.75) or poor (ICC <0.5) agreement. A glossary of measurement abbreviations is found in Table 2. Results are presented as RAD indicating those obtained with radiography and T1, T2* or STIR indicating those obtained with the relevant MRI sequence; m is added if a marker was used.

Abbreviations: Di, distal; Mi, mid; Pr, proximal.

**TABLE 4:** Inter‐modality comparison for each angle measurement (comparisons between measurements with poor repeatability and or excessive unattainable values are excluded) using an intra‐class correlation (ICC) with 95% confidence intervals (CI).

Angle	Comparison	Intraclass correlation	ICC rating	Lower confidence interval	Upper confidence interval	*p*
Coronet angle	*RAD* versus *RADm*	*0.80*	*Good*	*0.36*	*0.95*	*0.001*
	RAD versus T1	0.55	Moderate	−0.04	0.87	0.02
	RAD versus T2*	0.51	Moderate	−0.08	0.86	0.03
	*RADm* versus *T1m*	*0.79*	*Good*	*0.33*	*0.95*	*0.002*
	T1 versus T1m	0.61	Moderate	0.02	0.90	0.01
CP3ang	*RAD* versus *RADm*	*0.82*	*Good*	*0.39*	*0.96*	*<0.001*
	*RAD* versus *T1*	*0.81*	*Good*	*0.13*	*0.96*	*<0.001*
	*RAD* versus *STIR*	*0.81*	*Good*	*0.39*	*0.95*	*0.002*
DiP2ang	*RAD* versus *RADm*	*0.99*	*Excellent*	*0.94*	*1.0*	*<0.001*
	RAD versus T1	0.10	Poor	−0.56	0.69	0.4
	RAD versus T2*	0.06	Poor	−0.58	0.66	0.4
	RAD versus STIR	0.05	Poor	−0.60	0.66	0.4
DP3ang	*RAD* versus *RADm*	*0.97*	*Excellent*	*0.87*	*0.99*	*<0.001*
	RAD versus T1	0.2	Poor	−0.14	0.66	0.1
	RAD versus T2*	0.11	Poor	−0.17	0.57	0.3
	RAD versus STIR	0.04	Poor	−0.29	0.55	0.4
	RADm versus T1m	0.31	Poor	−0.19	0.76	0.1
	T1 versus T1m	0.29	Poor	−0.21	0.75	0.1
DiP3ang	*RAD* versus *RADm*	*0.91*	*Excellent*	*0.69*	*0.98*	*<0.001*
	RAD versus T1	0.19	Poor	−0.23	0.68	0.2
	RAD versus T2*	0.13	Poor	−0.17	0.60	0.2
	RAD versus STIR	0.13	Poor	−0.18	0.60	0.3
	RADm versus T1m	0.54	Moderate	−0.20	0.88	0.07
	RADm versus T2*m	0.52	Moderate	−0.19	0.87	0.07
	RADm versus STIRm	0.31	Poor	−0.49	0.79	0.2
	T1 versus T1m	0.24	Poor	−0.20	0.71	0.2
	T2*versus T2*m	0.05	Poor	−0.29	0.57	0.4
	STIR versus STIRm	0.09	Poor	−0.30	0.60	0.4
HCang	RAD versus RADm	0.82	Good	0.40	1.0	0.001
	RADm versus T1m	0.20	Poor	−0.37	0.72	0.3
	RADm versus T2*m	0.22	Poor	−0.33	0.72	0.2
PrP2ang	*RAD* versus *RADm*	*0.99*	*Excellent*	*0.95*	*1.0*	*<0.001*
	RAD versus T1	0.52	Moderate	−0.22	0.87	0.07
	RAD versus T2*	0.54	Moderate	−0.20	0.88	0.07
	RAD versus STIR	0.65	Moderate	0.02	0.91	0.03
	RADm versus T1m	0.41	Poor	−0.31	0.83	0.1
	T1 versus T1m	0.68	Moderate	0.14	0.92	0.01
Solar angle	RAD versus RADm	0.60	Moderate	−0.04	0.89	0.04
	RAD versus T1	0.04	Poor	−0.07	0.34	0.3
	RAD versus T2*	0.01	Poor	−0.10	0.32	0.4
	RAD versus STIR	0.07	Poor	−0.05	0.39	0.1
	RADm versus T1m	0.39	Poor	−0.12	0.81	0.02
	T1 versus T1m	0.21	Poor	−0.14	0.66	0.1

*Note*: Lines in italics highlight measurements with good (ICC 0.75–0.90) or excellent (ICC >0.9) agreement; we draw the reader's attention to the comparatively small number of measurements that met these criteria compared to measurements with moderate (ICC 0.5–0.75) or poor (ICC <0.5) agreement. A glossary of measurement abbreviations is found in Table 2. Results are presented as RAD indicating those obtained with radiography and T1, T2* or STIR indicating those obtained with the relevant MRI sequence; m is added if a marker was used.

#### 3.3.1 Laminitis measurements

For founder distance, there was poor to moderate agreement between plain radiographic and T1 and T2* measurements. There was good agreement between radiographic and T1 measurements with markers. Raw radiographic values were observed to be larger than MRI measurements. No comparisons were possible between radiographic and MRI measurements of P3rotang due to unacceptable repeatability for radiographic measurements with markers.

#### 3.3.2 Sagittal hoof wall measurements

Agreement between radiographic and MRI proximal measurements of hoof wall thickness and epidermal thickness was moderate to good. Comparisons between proximal dermal measurements were not possible.

There was variable agreement for sagittal hoof wall measurements in the mid dorsal hoof wall. Mid dorsal hoof wall thickness measurements had moderate agreement. Mid dorsal dermal thickness measurements had poor to moderate agreement for comparisons without markers but good to excellent agreement with markers. Agreement between radiographic and MRI measurements of mid dorsal epidermal thickness was poor.

There was also variable inter‐modality agreement for sagittal distal hoof wall measurements. Distal dorsal hoof wall thickness and epidermal thickness measurements had moderate to excellent agreement. Distal dorsal dermal thickness measurements ranged from poor to moderate without markers and poor to good with markers.

Radiographic measurements were often observed to be larger than corresponding MRI values before a ratio was applied.

#### 3.3.3 Solar thickness and toe length

Good agreement was seen between radiographic and MRI measurements with markers for sole thickness and epidermal sole thickness. Raw radiographic values were more often noted to be larger than MRI values. Comparisons of dermal sole thickness measurements were often not possible and, where possible, showed poor agreement. Moderate agreement was seen between radiographic and STIR measurements of toe length (with markers); raw radiographic measurements were observed to be larger.

#### 3.3.4 Frontal hoof wall measurements

Inter‐modality comparison was possible for a limited number of measurements and revealed generally poor agreement between radiographic and MRI measurements. More often, MRI measurements were larger than radiographic values before ratios were applied.

#### 3.3.5 Angles

Poor to moderate agreement was seen between radiographs and MRI for most angle measurements. Most sagittal angle measurements had larger values on MRI compared to radiographs. The relative sizes of MRI and radiographic frontal angles varied.

### 3.4 Hypothesis 3: The effect of hoof wall markers on foot measurements

#### 3.4.1 Radiographic hoof wall markers and unattainable values

The effect of radiographic markers on measurement attainability was variable for frontal parameters. Without radiographic markers, unattainable values were noted for some frontal epidermal and dermal parameters (specifically distal LDWT and LEWT and proximal LDWT and LEWT). For radiographic measurements with markers, unattainable values were noted for proximal lateral frontal epidermal and dermal parameters (LEWTm and LDWTm).

#### 3.4.2 Hoof wall ratio measurements

There was good to excellent agreement between radiographic measurements with and without markers for most parameters; exceptions were mid DHWT and proximal MEWT (moderate agreement) and proximal LEWT and mid DEWT (poor agreement).

Where possible, ICC for MRI measurements with and without the water‐soaked hoof bandage was poor to moderate.

#### 3.4.3 Angles

Agreement between radiographic angle measurements with and without markers was good to excellent, excepting solar angle (moderate ICC). Agreement between MRI measurements of DiP3ang with and without the WSHB was poor for all sequences. Additionally, ICC for T1 MRI measurements of DP3ang and solar angle was poor and moderate for coronet angle and PrP2ang.

## 4 DISCUSSION

The water‐soaked hoof bandage permitted visualisation of most of the hoof capsule except for its distal margins. There was limited agreement between radiographic and MRI measurements of equine hoof parameters, supporting the second hypothesis. Regarding the third hypothesis, there is evidence indicating good agreement between radiographic measurements with and without hoof wall markers but moderate to poor agreement between MRI values with and without the water‐soaked hoof bandage.

Intraobserver repeatability was at least ‘good’ for most measurements. A notable exception was frontal distal dermal measurements, which often had poor repeatability for radiographs and MRI. On radiographs, poor definition between the strata media and interna likely explains this, despite the use of windowing. On MRI, a clear border between the two structures was not always evident. A previous high‐field study reported that MRI measurements had better intraobserver variability compared to radiographs.^4^ This was the opposite in our study, perhaps reflecting greater image detail in high‐field MRI.^14^

### 4.1 Hypothesis 1: Use of the water‐soaked hoof bandage

The water‐soaked hoof bandage was a simple method to visualise the hoof capsule during MRI; however, some distal measurements were not consistently possible. Inability to visualise the hoof margins could be due to distal structures being at the edge of the field of view with associated field inhomogeneities and gradient non‐linearities such as seen in Figure 1C,D. This has previously been reported in low‐field and not in high‐field MRI.^15^ Other possible causes are the need for a thicker bandage or radiographically invisible metallic shoe clenches causing susceptibility artefact.^16^ While the use of a water‐soaked bandage is reported,^11^ this is the first study to assess the method's limitations, which is important for application in clinical practice.

### 4.2 Hypothesis 2: Agreement between MRI and radiographic foot measurements

Agreement between radiographic and MRI foot measurements was limited; it was not possible to identify one MRI sequence as having the best agreement with radiographs compared to other sequences. A similar low‐field MRI study found significant differences between imaging sources for a limited series of hoof measurements.^8^ A possible cause of this discrepancy is the inherent nature of these imaging modalities, with radiographs being 2D images and MRI producing 3D images. This could cause measurement variation despite a strict protocol. Also in MRI, compensation for limb position through piloting potentially alters perception of the hoof's relationship with the ground.^8^

For sagittal measurements, radiographic values often exceeded MRI values. A previous high‐field MRI study^2^ noted similar findings for founder distance and dorsal hoof wall thickness. A recent cadaver study also found larger sagittal radiographic values compared to measurements from computed tomography (CT) images.^17^ Another high‐field study found good inter‐modality agreement for dorsal hoof wall measurements but poorer agreement for frontal hoof capsule measurements with smaller MRI values compared to radiographs.^4^ The present study found better, albeit variable, inter‐modality agreement for dorsal hoof wall measurements, whereas agreement for frontal measurements was consistently poor. Frontal MRI values exceeded radiographic values in the current study. Differences between high and low‐field studies could be due to greater gradient non‐linearity and magnetic field inhomogeneity in low‐field systems causing greater image distortion.^18^ The reason for the differing relative sizes of frontal and sagittal plane measurements is unclear; the curvature of the hoof capsule may result in greater radiographic summation in the sagittal compared to the frontal plane. The limited agreement between the modalities indicates that MRI hoof measurements cannot be used as a substitute for radiographic assessment. Potential exceptions are sole and epidermal sole thickness measurements which showed good agreement. Future studies could aim to establish a consistent relationship between radiographic and MRI foot measurements to improve MRI assessment of the foot.

Generally, measurements in our study were smaller than comparable measurements of draft breeds^13^ and Warmbloods (excepting P3rotang),^5^ and larger than those reported in ponies (excepting founder distance).^19^ Our study aimed to compare imaging modalities, and given that the breed and farriery of the studied cadavers was unknown, these data do not serve as reference values.

### 4.3 Hypothesis 3: The effect of hoof wall markers on foot measurements

Radiographic dorsal hoof wall markers were associated with below threshold repeatability compared to plain measurements for P3rotang; poorer repeatability was also noted for distal DDWT, proximal DDWT, and proximal DEWT, which are used in laminitis assessment.^20^ Our findings suggest that radiographic hoof wall markers may not provide acceptably repeatable measurements. Poor repeatability could be due to divergence of the hoof wall marker from the hoof capsule or obscuration of the hoof capsule by the marker, likely due to the Mach phenomenon.^21^ Variations in marker placement have also been reported,^6^ and are likely more significant in founder distance assessment. In our study, there was no difference in founder distance repeatability with and without hoof wall markers, and good to excellent agreement was noted between most radiographic measurements with and without markers. These findings support previous research that radiographic markers offer no benefit,^9^ and may hinder radiographic interpretation.

In some instances, the water‐soaked hoof bandage appeared to affect MRI measurement repeatability. Frontal phalangeal angles had good repeatability without but poor repeatability with the water‐soaked hoof bandage. This could be due to the bandage not following the contour of the sole correctly; a ‘line of best fit’ was often necessary to perform this measurement. A similar pattern was not noted for sagittal angle measurements. Agreement between values obtained from plain MRI and those with the water‐soaked hoof bandage was poor to moderate, suggesting that the bandage substantially alters the measurements acquired.

## 5 LIMITATIONS

The extensive measurements performed in this study limited the number of limbs feasible hence a small convenience sample was used. More nuanced conclusions with narrower confidence intervals might be found with greater numbers. The signalment and clinical history of the horses was unknown therefore lameness cannot be definitively excluded. A marker of known size was not used to account for radiographic magnification but used P3L as a reference instead. Use of ratios is more practical in the field given it precludes the need for a marker. The measurements performed had generally good intra‐observer repeatability however interobserver repeatability was not investigated. The study used cadavers rather than live horses for practicality hence the current findings may not apply to limbs with pathology,^2^ nor loaded limbs due to potential hoof capsule distortion and altered alignment of the phalangeal column.^22^ The use of ratios likely mitigated this, and each foot maintained a comparable stance between image acquisitions. One cadaver study found dorsal hoof thicknesses obtained using digital radiography to either be slightly larger or similar to measurements from studies in live horses.^4^ Application of hoof wall markers may also be more challenging with hoof capsules that vary in shape and can vary between operators.^6^ A ‘gold standard’ comparison was lacking hence the present study cannot define radiography or MRI as providing more accurate measurements than the other. Interestingly, a recent study found CT measurements showed greater agreement with gross pathological measurements compared to radiographs.^17^

## 6 CONCLUSION

In conclusion, low‐field MRI foot measurements showed limited agreement with radiographic values. This is clinically important, as low‐field systems are commonly used in equine practice; contemporaneous radiographs are recommended to provide a more complete assessment of the foot compared to MRI alone. Radiographic markers offer no benefit and can potentially hinder hoof assessment. A water‐soaked hoof bandage can visualise most of the hoof capsule during MRI, but at present, cannot be recommended to aid hoof parameter measurements. Hoof wall markers should be used with caution in practice.

## FUNDING INFORMATION

Not applicable.

## CONFLICT OF INTEREST STATEMENT

The authors declare no conflicts of interest.

## AUTHOR CONTRIBUTIONS

**David M. Bolt:** Conceptualization; writing – review and editing; formal analysis; supervision; methodology. **Yu‐Mei Chang:** Formal analysis; supervision; writing – review and editing. **Dagmar Berner:** Conceptualization; investigation; methodology; supervision; writing – review and editing; formal analysis. **Constance Bowkett‐Pritchard:** Investigation; writing – original draft; writing – review and editing; formal analysis; project administration; visualization.

## DATA INTEGRITY STATEMENT

Constance Bowkett‐Pritchard had full access to all the data in the study and takes responsibility for the integrity of the data and the accuracy of the data analysis.

## ETHICAL ANIMAL RESEARCH

Research ethics committee oversight not currently required by this journal: the study was performed on archived material collected previously during clinical procedures.

## INFORMED CONSENT

Not stated.

## ANTIMICROBIAL STEWARDSHIP POLICY

Not applicable.

## Supporting information


**Table S1:** Detailed instructions regarding how the measurements described were performed using digital imaging software (Horos).

**Table S2.** (a) Descriptive statistics for sagittal hoof wall measurements ratio and modality; mean and standard deviation (SD) are presented. (b) Intraobserver reliability (intra‐class correlation; ICC) for each measurement ratio and modality.

**Table S3.** (a) Descriptive statistics for each dorsal hoof wall measurement ratio and modality; mean and standard deviation (SD) are presented. (b) Intraobserver reliability (intra‐class correlation; ICC) for each measurement ratio and modality.

**Table S4.** (a) Descriptive statistics for each angle measurement and modality; mean and standard deviation (SD) are presented. (b) Intraobserver reliability (intra‐class correlation; ICC) for each measurement ratio and modality.

## Data Availability

The data that support the findings of this study are openly available at https://data.mendeley.com/datasets/fwppz4jpk2/1.
