# Supplementary material for: Measurements of equine foot parameters show limited agreement between radiographs and low‐field magnetic resonance imaging
Source: Equine Vet J. 2025 Jun 26;57(5):1231–44. doi: 10.1111/evj.14536 (PMC12326914; doi:10.1111/evj.14536)
Supplement: Supplementary file 2 — Table S2. (a) Descriptive statistics for sagittal hoof wall measurements ratio and modality; mean and standard deviation (SD) are presented. (b) Intraobserver reliability (intra‐class correlation; ICC) for each measurement ratio and modality. [file EVJ-57-1231-s004.pdf]

Equine Veterinary Journal
BEVA

Table S2a: Descriptive statistics for sagittal hoof wall measurements ratio and modality; mean and standard deviation (SD) are presented. Note P3rotang is a raw value. Comparisons not possible between plain radiographs and MRI are indicated n/a.

**Table S2a Measurement**	**Modality and Descriptive Statistics**															
	**RAD**		**RADm**		**T1**		**T1m**		**T2***		**T2*m**		**STIR**		**STIRm**	
	**Mean**	**SD**	**Mean**	**SD**	**Mean**	**SD**	**Mean**	**SD**	**Mean**	**SD**	**Mean**	**SD**	**Mean**	**SD**	**Mean**	**SD**
	Dorsal Hoof Wall Measurements															
DiDDWT	0.10	0.01	0.10	0.02	0.10	0.02	0.11	0.02	0.08	0.02	0.09	0.02	0.08	0.02	0.08	0.02
DiDEWT	0.13	0.023	0.13	0.02	n/a	n/a	0.12	0.02	n/a	n/a	0.12	0.02	n/a	n/a	0.12	0.03
DiDHWT	0.23	0.03	0.23	0.03	n/a	n/a	0.22	0.03	n/a	n/a	0.21	0.04	n/a	n/a	0.20	0.04
MiDDWT	0.09	0.02	0.09	0.02	0.09	0.01	0.10	0.02	0.09	0.01	0.09	0.02	0.09	0.01	0.09	0.02
MiDEWT	0.15	0.02	0.14	0.02	n/a	n/a	0.14	0.02	n/a	n/a	0.15	0.02	n/a	n/a	0.14	0.02
MiDHWT	0.24	0.03	0.23	0.02	n/a	n/a	0.24	0.03	n/a	n/a	0.24	0.03	n/a	n/a	0.23	0.03
PrDDWT	0.10	0.01	0.13	0.01	0.11	0.01	0.11	0.02	0.11	0.01	0.11	0.01	0.11	0.01	0.11	0.01
PrDEWT	0.15	0.02	0.15	0.02	n/a	n/a	0.16	0.03	n/a	n/a	0.16	0.03	n/a	n/a	0.15	0.02
PrDHWT	0.25	0.03	0.25	0.03	n/a	n/a	0.27	0.03	n/a	n/a	0.27	0.03	n/a	n/a	0.26	0.03
	Solar Measurements															
DST	0.07	0.02	0.07	0.02	0.09	0.09	0.09	0.01	0.08	0.02	0.08	0.01	0.08	0.02	0.08	0.02
EST	0.12	0.03	0.12	0.03	n/a	n/a	0.12	0.04	n/a	n/a	0.12	0.03	n/a	n/a	0.12	0.03
Sole thickness	0.19	0.04	0.19	0.05	n/a	n/a	0.22	0.04	n/a	n/a	0.19	0.04	n/a	n/a	0.19	0.05
Toe length	0.47	0.10	0.45	0.10	n/a	n/a	0.40	0.11	n/a	n/a	0.36	0.13	n/a	n/a	0.39	0.14
	Laminitis Measurements															
Founder distance	0.10	0.02	0.09	0.03	0.08	0.02	0.08	0.03	0.08	0.02	0.09	0.02	0.08	0.02	0.07	0.02
P3rotang	2.51	1.20	2.72	1.10	n/a	n/a	2.83	1.13	n/a	n/a	3.52	1.53	n/a	n/a	5.17	1.10

Table S2b: Intraobserver reliability (intra-class correlation; ICC) for each measurement ratio and modality. Lower (LCI) and upper (UCI) confidence intervals are presented. Statistical significance is p < 0.05. * <5 unattainable values, ** ≥5 unattainable values.

Table S2b Measurement	Intraobserver Repeatability															
	RAD		RADm		T1		T1m		T2*		T2*m		STIR		STIRm	
	ICC *(LCI-UCI)*	p value	ICC *(LCI-UCI)*	p value	ICC *(LCI-UCI)*	p value	ICC *(LCI-UCI)*	p value	ICC *(LCI-UCI)*	p value	ICC *(LCI-UCI)*	p value	ICC *(LCI-UCI)*	p value	ICC *(LCI-UCI)*	p value
	Dorsal Hoof Wall Measurements															
DiDDWT	0.85 *(0.63-0.96)*	<0.001	0.78 *(0.45-0.94)*	<0.001	0.96 *(0.89-0.99)*	<0.001	0.97 *(0.92-0.99)*	<0.001	0.92* *(0.77-0.98)*	<0.001	0.97 *(0.92-0.99)*	<0.001	0.86 *(0.65-0.96)*	<0.001	0.96 *(0.89-0.99)*	<0.001
DiDEWT	0.57 *(0.18-0.87)*	0.003	0.97 *(0.92-0.99)*	<0.001	n/a	n/a	0.97 *(0.86-0.99)*	<0.001	n/a	n/a	0.92 *(0.74-0.98)*	<0.001	n/a	n/a	0.95 *(0.85-0.99)*	<0.001
DiDHWT	>0.99 *(0.99-1.0)*	<0.001	0.99 *(0.97-1.0)*	<0.001	n/a	n/a	0.99 *(0.95-1.0)*	<0.001	n/a	n/a	0.97 *(0.88-0.99)*	<0.001	n/a	n/a	0.98 *(0.94-1.0)*	<0.001
MiDDWT	0.91 *(0.74-0.98)*	<0.001	0.92 *(0.79-0.98)*	<0.001	0.90 *(0.72-0.97)*	<0.001	0.96 *(0.88-0.99)*	<0.001	0.96* *(0.88-0.99)*	<0.001	0.92 *(0.78-0.98)*	<0.001	0.80 *(0.52-0.94)*	<0.001	0.87 *(0.66-0.97)*	<0.001
MiDEWT	0.97 *(0.90-0.99)*	<0.001	0.91 *(0.72-0.98)*	<0.001	n/a	n/a	0.97 *(0.91-0.99)*	<0.001	n/a	n/a	0.97 *(0.90-0.99)*	<0.001	n/a	n/a	0.96 *(0.87-0.99)*	<0.001
MiDHWT	0.99 *(0.97-1.0)*	<0.001	0.99 *(0.98-1.0)*	<0.001	n/a	n/a	0.99 *(0.96-1.0)*	<0.001	n/a	n/a	0.98 *(0.95-1.0)*	<0.001	n/a	n/a	0.93 *(0.91-0.98)*	<0.001
PrDDWT	0.64 *(0.27-0.89)*	<0.001	0.43 *(-0.01-0.81)*	0.03	0.29 *(-0.04-0.71)*	0.05	0.88 *(0.63-0.97)*	<0.001	0.85 *(0.61-0.96)*	<0.001	0.83 *(0.49-0.96)*	<0.001	0.59 *(0.20-0.87)*	0.002	0.77 *(0.46-0.94)*	<0.001
PrDEWT	0.98 *(0.93-0.99)*	<0.001	0.80 *(0.51-0.95)*	<0.001	n/a	n/a	0.95 *(0.87-0.99)*	<0.001	n/a	n/a	0.75 *(0.34-0.93)*	<0.001	n/a	n/a	0.98 *(0.92-0.99)*	<0.001
PrDHWT	0.97 *(0.90-0.99)*	<0.001	0.97 *(0.90-0.99)*	<0.001	n/a	n/a	0.98 *(0.93-0.99)*	<0.001	n/a	n/a	0.98 *(0.93-0.99)*	<0.001	n/a	n/a	0.98 (0.92-1.0	<0.001
	Solar Measurements															
DST	0.79 *(0.50-0.94)*	<0.001	0.78 *(0.49-0.94)*	<0.001	0.25 *(-0.16-0.71)*	0.1	0.26 *(-0.06-0.69)*	0.06	0.56 *(0.16-0.86)*	0.004	0.55 *(0.17-0.85)*	0.002	0.77 *(0.45-0.94)*	<0.001	-0.29 *-0.46-0.16)*	0.9
EST	0.76 *(0.44-0.93)*	<0.001	0.98 *(0.94-1.00)*	<0.001	n/a	n/a	0.85 *(0.61-0.96)*	<0.001	n/a	n/a	0.83 *(0.53-0.96)*	<0.001	n/a	n/a	0.97 *(0.90-0.99)*	<0.001
Sole thickness	0.96 *(0.88-0.99)*	<0.001	0.98 *(0.93-0.99)*	<0.001	n/a	n/a	0.77 *(0.47-0.94)*	<0.001	n/a	n/a	0.97 *(0.90-0.99)*	<0.001	n/a	n/a	0.99 *(0.96-1.00)*	<0.001
Toe length	0.99 *(0.96-1.0)*	<0.001	0.97 *(0.90-.99)*	<0.001	n/a	n/a	0.98** *(0.83-1.0)*	<0.001	n/a	n/a	0.99** *(0.85-1.0)*	<0.001	n/a	n/a	0.98 *(0.93-0.99)*	<0.001
	Laminitis Measurements															
Founder distance	0.91 *(0.57-0.98)*	<0.001	0.98 *(0.94-1.00)*	<0.001	0.87 *(0.64-0.97)*	<0.001	0.77 *(0.38-0.94)*	<0.001	0.77 *(0.30-0.94)*	<0.001	0.45 *(0.08-0.81)*	0.008	0.19 *(-0.09-0.63)*	0.1	0.20 *(-0.19-0.68)*	0.2
P3rotang	0.77 *(0.47-0.94)*	<0.001	0.62 *(0.22-0.89)*	<0.001	n/a	n/a	0.64 *(0.27-0.89)*	<0.001	n/a	n/a	0.76 *(0.44-0.93)*	<0.001	n/a	n/a	0.39 *(0.00-0.78)*	0.03

A glossary of measurement abbreviations is found in Table 2. Results are presented as RAD indicating those obtained with radiography and T1, T2* or STIR indicating those obtained with the relevant MRI sequence; m is added if a marker was used. Further abbreviations are: Di distal; Mi mid; Pr proximal.
