# Supplementary material for: Measurements of equine foot parameters show limited agreement between radiographs and low‐field magnetic resonance imaging
Source: Equine Vet J. 2025 Jun 26;57(5):1231–44. doi: 10.1111/evj.14536 (PMC12326914; doi:10.1111/evj.14536)
Supplement: Supplementary file 3 — Table S3. (a) Descriptive statistics for each dorsal hoof wall measurement ratio and modality; mean and standard deviation (SD) are presented. (b) Intraobserver reliability (intra‐class correlation; ICC) for each measurement ratio and modality. [file EVJ-57-1231-s003.pdf]

Table S3a: Descriptive statistics for each dorsal hoof wall measurement ratio and modality; mean and standard deviation (SD) are presented. Comparisons not possible between plain radiographs and MRI are indicated n/a.

Table S3a Measurement	Modality and Descriptive Statistics															
	RAD		RADm		T1		T1m		T2*		T2*m		STIR		STIRm	
	Mean	SD	Mean	SD	Mean	SD	Mean	SD	Mean	SD	Mean	SD	Mean	SD	Mean	SD
DiLDWT	0.11	0.02	0.12	0.03	0.13	0.03	0.15	0.02	0.16	0.04	0.14	0.03	0.19	0.05	0.14	0.02
DiLEWT	0.12	0.02	0.12	0.02	n/a	n/a	0.17	0.04	n/a	n/a	0.14	0.03	n/a	n/a	0.14	0.03
DiLHWT	0.24	0.02	0.23	0.02	n/a	n/a	0.31	0.05	n/a	n/a	0.29	0.04	n/a	n/a	0.30	0.04
DiMDWT	0.14	0.02	0.13	0.02	0.17	0.03	0.15	0.04	0.16	0.03	0.14	0.03	0.20	0.05	0.16	0.05
DiMEWT	0.12	0.02	0.11	0.03	n/a	n/a	0.13	0.02	n/a	n/a	0.15	0.04	n/a	n/a	0.13	0.05
DiMHWT	0.25	0.04	0.25	0.024	n/a	n/a	0.30	0.04	n/a	n/a	0.30	0.03	n/a	n/a	0.29	0.05
PrLDWT	0.11	0.03	0.12	0.03	0.23	0.02	0.22	0.02	0.25	0.02	0.24	0.02	0.20	0.03	0.22	0.01
PrLEWT	0.14	0.02	0.12	0.03	n/a	n/a	0.17	0.02	n/a	n/a	0.16	0.03	n/a	n/a	0.14	0.02
PrLHWT	0.26	0.04	0.24	0.04	n/a	n/a	0.39	0.03	n/a	n/a	0.40	0.03	n/a	n/a	0.36	0.03
PrMDWT	0.10	0.02	0.10	0.02	0.21	0.02	0.21	0.03	0.23	0.02	0.23	0.02	0.20	0.02	0.20	0.02
PrMEWT	0.13	0.02	0.13	0.03	n/a	n/a	0.16	0.02	n/a	n/a	0.15	0.02	n/a	n/a	0.14	0.02
PrMHWT	0.24	0.03	0.23	0.04	n/a	n/a	0.38	0.03	n/a	n/a	0.38	0.03	n/a	n/a	0.34	0.03

Table S3b: Intraobserver reliability (intra-class correlation; ICC) for each measurement ratio and modality. Lower (LCI) and upper (UCI) confidence intervals are presented. Statistical significance is $p < 0.05$. * <5 unattainable values, ** ≥5 unattainable values.

**Table S3b Measurement**	**Intraobserver Repeatability**															
	**RAD**		**RADm**		**T1**		**T1m**		**T2***		**T2*m**		**STIR**		**STIRm**	
	**ICC** ***(LCI-UCI)***	**p value**	**ICC** ***(LCI-UCI)***	**p value**	**ICC** ***(LCI-UCI)***	**p value**	**ICC** ***(LCI-UCI)***	**p value**	**ICC** ***(LCI-UCI)***	**p value**	**ICC** ***(LCI-UCI)***	**p value**	**ICC** ***(LCI-UCI)***	**p value**	**ICC** ***(LCI-UCI)***	**p value**
DiLDWT	0.72* *(0.36-0.93)*	<0.001	0.52 *(0.14-0.84)*	0.005	0.35* *(-0.09-0.84)*	0.08	0.02 *(-0.21-0.48)*	0.42	-0.21** *(-0.75-0.91)*	0.59	0.38 *(-0.03-0.78)*	0.04	0.92 *(0.74-0.98)*	<0.001	-0.02* *(-0.33-0.54)*	<0.001
DiLEWT	0.85* *(0.61-0.97)*	<0.001	0.64 *(0.28-0.89)*	<0.001	n/a	n/a	0.85* *(0.46-0.98)*	<0.001	n/a	n/a	0.82* *(0.50-0.96)*	<0.001	n/a	n/a	0.75* *(0.41-0.94)*	<0.001
DiLHWT	0.82 *(0.56-0.95)*	<0.001	0.46 *(0.08-0.81)*	0.009	n/a	n/a	0.87* *(0.57-0.98)*	<0.001	n/a	n/a	0.64* *(0.19-0.92)*	0.004	n/a	n/a	0.41 *(-0.00-0.80)*	0.03
DiMDWT	0.74 *(0.38-0.93)*	<0.001	*0.51* (0.13-0.84)	0.004	0.67** *(0.08-0.97)*	0.001	0.53 *(0.08-0.90)*	0.008	0.54** *(-0.02-0.96)*	0.05	0.45* *(0.02-0.85)*	0.03	0.87* *(0.62-0.97)*	<0.001	0.52* *(0.08-0.86)*	0.01
DiMEWT	0.93 *(0.81-0.98)*	<0.001	0.98 *(0.92-0.99)*	<0.001	n/a	n/a	-0.06** *(-1.0-1.0)*	0.45	n/a	n/a	0.62* *0.03-0.95)*	0.02	n/a	n/a	0.90* *(0.71-0.98)*	<0.001
DiMHWT	0.93 *(0.81-0.98)*	<0.001	*0.95* *(0.85-0.99)*	<0.001	n/a	n/a	0.84** *(0.22-1.0)*	0.02	n/a	n/a	0.33* *(-0.11-0.87)*	0.10	n/a	n/a	0.87 *(0.65-0.97)*	<0.001
PrLDWT	0.59* *(0.15-0.89)*	0.005	0.97* *(0.90-0.99)*	<0.001	0.80 *(0.40-0.95)*	<0.001	*0.90* *(0.74-0.97)*	<0.001	0.87 *(0.65-0.96)*	<0.001	0.58 *(0.19-0.87)*	<0.001	0.43 *(0.05-0.79)*	0.005	0.06 *(-0.26-0.56)*	0.4
PrLEWT	0.80* *(0.50-0.95)*	<0.001	0.90* *(0.71-0.98)*	<0.001	n/a	n/a	0.80 *(0.44-0.95)*	<0.001	n/a	n/a	0.69 *(0.34-0.91)*	<0.001	n/a	n/a	0.84 *(0.57-0.96)*	0.6
PrLHWT	0.83 *(0.58-0.95)*	<0.001	0.96 *(0.88-0.99)*	<0.001	n/a	n/a	0.97 *(0.93-0.99)*	<0.001	n/a	n/a	0.99 *(0.96-1.00)*	<0.001	n/a	n/a	0.58 *(0.20-0.86)*	0.001
PrMDWT	0.82 *(0.54-0.95)*	<0.001	0.76 *(-0.44-0.93)*	<0.001	0.97 *(0.92-0.93)*	<0.001	0.86 *(0.63-0.96)*	<0.001	0.88 *(0.66-0.97)*	<0.001	0.88 *(0.68-0.97)*	<0.001	0.83 *(0.52-0.96)*	<0.001	0.68 *(0.32-0.90)*	<0.001
PrMEWT	0.91 *(0.76-0.98)*	<0.001	0.87 *(0.64-0.97)*	<0.001	n/a	n/a	0.79 *(0.50-0.94)*	<0.001	n/a	n/a	0.66 *(0.29-0.90)*	<0.001	n/a	n/a	0.93 *(0.80-0.98)*	<0.001
PrMHWT	0.95 *(0.85-0.99)*	<0.001	0.98 *(0.93-0.99)*	<0.001	n/a	n/a	0.95 *(0.86-0.99)*	<0.001	n/a	n/a	0.95 *(0.85-0.99)*	<0.001	n/a	n/a	0.83 *(0.58-0.95)*	<0.001

A glossary of measurement abbreviations is found in Table 2. Results are presented as RAD indicating those obtained with radiography and T1, T2* or STIR indicating those obtained with the relevant MRI sequence; m is added if a marker was used. Further abbreviations are: Di distal; Mi mid; Pr proximal.
