# Supplementary material for: Measurements of equine foot parameters show limited agreement between radiographs and low‐field magnetic resonance imaging
Source: Equine Vet J. 2025 Jun 26;57(5):1231–44. doi: 10.1111/evj.14536 (PMC12326914; doi:10.1111/evj.14536)
Supplement: Supplementary file 4 — Table S4. (a) Descriptive statistics for each angle measurement and modality; mean and standard deviation (SD) are presented. (b) Intraobserver reliability (intra‐class correlation; ICC) for each measurement ratio and modality. [file EVJ-57-1231-s001.pdf]

Equine Veterinary Journal BEVA

Table S4a: Descriptive statistics for each angle measurement and modality; mean and standard deviation (SD) are presented. Comparisons not possible between plain radiographs and MRI are indicated n/a.

Table S4a Measurement	Modality and Descriptive Statistics															
	RAD		RADm		T1		T1m		T2*		T2*m		STIR		STIRm	
	Mean	SD	Mean	SD	Mean	SD	Mean	SD	Mean	SD	Mean	SD	Mean	SD	Mean	SD
Coronet angle	23.76	3.59	24.96	3.57	21.41	3.65	23.68	4.25	21.32	3.26	23.84	3.53	21.40	2.96	24.45	3.63
CP3ang	70.14	3.63	71.36	3.76	71.70	2.92	71.2	3.48	71.25	2.37	70.97	2.54	70.86	3.43	70.04	2.83
DiP2ang	2.20	1.53	2.07	1.52	1.52	1.51	2.17	1.03	1.47	1.54	2.12	1.21	1.49	1.53	2.66	0.67
DiP3ang	19.06	1.49	18.86	1.54	21.13	2.53	18.81	2.18	21.97	2.60	19.27	2.19	21.86	2.58	19.11	2.61
DP3ang	46.23	1.79	6.12	1.70	49.59	2.76	47.60	1.73	49.33	2.45	46.75	1.67	49.09	3.0	45.73	1.49
HCang	48.85	1.70	49.30	1.66	n/a	n/a	50.57	2.28	n/a	n/a	50.82	2.66	n/a	n/a	51.14	2.39
PrP2ang	3.17	1.85	3.18	1.77	3.41	2.47	2.59	2.19	3.15	2.02	3.11	2.24	2.82	2.01	2.80	1.63
Solar angle	3.27	1.64	2.82	1.67	7.61	1.55	5.08	1.96	7.75	2.02	5.55	1.60	8.90	2.03	5.57	2.19

Table S4b: Intraobserver reliability (intra-class correlation; ICC) for each measurement ratio and modality. Lower (LCI) and upper (UCI) confidence intervals are presented. Statistical significance is $p < 0.05$.

**Table S4b Measurement**	**Intraobserver Repeatability**															
	**RAD**		**RADm**		**T1**		**T1m**		**T2***		**T2*m**		**STIR**		**STIRm**	
	**ICC** ***(LCI-UCI)***	**p value**	**ICC** ***(LCI-UCI)***	**p value**	**ICC** ***(LCI-UCI)***	**p value**	**ICC** ***(LCI-UCI)***	**p value**	**ICC** ***(LCI-UCI)***	**p value**	**ICC** ***(LCI-UCI)***	**p value**	**ICC** ***(LCI-UCI)***	**p value**	**ICC** ***(LCI-UCI)***	**p value**
Coronet angle	0.80 *(0.52-0.94)*	<0.001	>0.99 *(0.99-1.00)*	<0.001	0.81 *0.54-0.95)*	<0.001	0.76 *(0.42-0.94)*	<0.001	0.88 *(0.68-0.97)*	<0.001	0.64 *(0.25-0.89)*	<0.001	0.67 *(-0.32-0.90)*	<0.001	0.66 *(0.30-0.90)*	<0.001
CP3ang	0.73 *(0.40-0.92)*	<0.001	0.97 *(0.92-0.99)*	<0.001	0.73 *(0.40-0.92)*	<0.001	0.68 *(0.17-0.91)*	<0.001	0.53 *(0.14-0.85)*	0.001	0.42 *(0.02-0.79)*	<0.001	0.71 *(0.37-0.92)*	<0.001	0.53 *(0.14-0.85)*	<0.001
DiP2ang	0.96 *(0.88-0.99)*	<0.001	0.98 *(0.95-1.00)*	<0.001	0.96 *(0.88-0.99)*	<0.001	0.23 *(-0.14-0.69)*	0.1	0.92 *(0.76-0.98)*	<0.001	0.28 *(-0.03-0.70)*	0.04	0.93 *(0.81-0.98)*	<0.001	-0.16 *(-0.29-0.21)*	0.9
DiP3ang	0.89 *(0.55-0.97)*	<0.001	0.89 *(0.67-0.97)*	<0.001	0.93 *(0.88-0.98)*	<0.001	0.88 *(0.69-0.97)*	<0.001	0.93 *(0.80-0.98)*	<0.001	0.77 *(0.46-0.94)*	<0.001	0.93 *(0.81-0.98)*	<0.001	0.80 *(0.52-0.94)*	<0.001
DP3ang	0.92 *(0.78-0.98)*	<0.001	0.89 *(0.70-0.97)*	<0.001	0.89 *(0.70-0.97)*	<0.001	0.74 *(0.40-0.93)*	<0.001	0.87 *(0.67-0.97)*	<0.001	0.58 *(0.18-0.87)*	0.003	0.93 *(0.81-0.98)*	<0.001	0.40 *(-0.01-0.79)*	0.03
HCang	0.92 *(0.79-0.98)*	<0.001	0.73 *(0.40-0.92)*	<0.001	n/a	n/a	0.88 *(0.68-0.97)*	<0.001	n/a	n/a	0.88 *(0.68-0.97)*	<0.001	n/a	n/a	0.52 *(0.13-0.84)*	0.005
PrP2ang	0.97 *(0.92-0.99)*	<0.001	0.97 *(0.92-0.99)*	<0.001	0.98 *(0.95-1.0)*	<0.001	0.85 *(0.62-0.96)*	<0.001	0.93 *(0.80-0.98)*	<0.001	0.55 *(0.16-0.85)*	0.004	0.89 *(0.70-0.97)*	<0.001	0.25 *(-0.10-0.69)*	0.1
Solar angle	0.77 *(0.46-0.94)*	<0.001	0.88 *(0.68-0.97)*	<0.001	0.93 *(0.80-0.98)*	<0.001	0.79 *(0.50-0.94)*	<0.001	0.94 *(0.84-0.99)*	<0.001	0.94 *(0.84-0.99)*	<0.001	0.93 *(0.81-0.98)*	<0.001	0.44 *(0.06-0.80)*	0.01

A glossary of measurement abbreviations is found in Table 2. Results are presented as RAD indicating those obtained with radiography and T1, T2* or STIR indicating those obtained with the relevant MRI sequence; m is added if a marker was used.
