# Supplementary material for: Measurements of equine foot parameters show limited agreement between radiographs and low‐field magnetic resonance imaging
Source: Equine Vet J. 2025 Jun 26;57(5):1231–44. doi: 10.1111/evj.14536 (PMC12326914; doi:10.1111/evj.14536)
Supplement: Supplementary file 1 — Table S1: Detailed instructions regarding how the measurements described were performed using digital imaging software (Horos). [file EVJ-57-1231-s002.pdf]

Equine Veterinary Journal BEVA

Table S1:

For sagittal MR measurements, align the sagittal image as close to halfway across the phalangeal column as possible.

For frontal MR measurements, the phalangeal column should be fully visible with the vascular channels in mid P3 clearly distinguishable.

Measurement	Description
Coronet Angle	Use dynamic angle function.<br>Line 1<br>• Coronary band<br>- Radiographs:<br>○ Without markers: proximal third of coronary band<br>○ With markers: proximal aspect of marker on coronary band<br>- MRI:<br>○ Without markers: proximal third of coronary band<br>○ With markers: narrowest point between bandage and coronary band<br>• Heel bulb<br>- Radiographs<br>○ Without markers: angle of heel bulb (there is a change in opacity in this region)<br>○ With markers: mid dorsal aspect of ball bearing<br>- MRI:<br>○ Palmar junction between digital cushion and frog<br>Line 2<br>• Radiographs: foot block<br>• MRI: horizontal guideline (plain images) OR line of best fit following solar surface of bandage (with WSHB).
Coronet to Distal Phalanx Angle (CP3ang)	Use dynamic angle function:<br>Line 1<br>• As for coronet angle<br>Line 2<br>• Dorsal P3
Distal Middle Phalanx Angle (DiP2ang)	Use dynamic angle function:<br>Line 1

	• Joins the flattest points of the distal condyles of P2<br>Line 2<br>• Radiographs: foot block<br>• MRI: horizontal guideline (plain images) OR line of best fit following solar surface of bandage (with WSHB)
Distal Third Phalanx Angle (DiP3ang)	Use dynamic angle function.<br>Line 1<br>• Facies solearis of P3<br>Line 2<br>• Radiographs: foot block<br>• MRI: horizontal guideline (plain images) OR line of best fit along solar surface of bandage (with WSHB)
Dorsal Third Phalanx Angle (DP3ang)	Use dynamic angle function.<br>Line 1<br>• Dorsal P3<br>Line 2<br>• Radiographs: foot block<br>• MRI: horizontal guideline (plain images) OR line of best fit along solar surface of bandage (with WSHB)
Dermal sole thickness (DST)	Points of reference:<br>• Dorsodistal tip of P3<br>• Dermal-epidermal solar junction<br>Measure vertical distance between the reference points.
Epidermal Sole Thickness (EST)	Reference Point:<br>• Sole level with dorsodistal P3 (barium sulphate line/weight-bearing surface of bandage)<br>• Dermal-epidermal solar junction<br>Measure vertical distance between the reference points.
Founder Distance	Reference Points:<br>• Dorsoproximal P3<br>AND<br>• Radiograph/MRI without marker: proximal third of the coronary band<br>• Radiographs with marker: proximal end of marker adjacent to coronary band<br>• MRI with marker: narrowest point between the bandage and the dorsoproximal coronary band<br>Draw a horizontal line from the reference point on the coronary band.<br>Measure a perpendicular line from the dorsoproximal aspect of the P3 extensor process to meet the horizontal line.
Hoof Capsule Angle (HCang)	Use dynamic angle function:<br>Line 1<br>• Dorsal hoof capsule

	Line 2<br>• Radiographs: foot block<br>• MRI: horizontal guideline (plain images) OR line of best fit following solar surface of bandage (with WSHB).
Distal Phalanx Length (P3L)	Distance from dorsodistal P3, following the facies solearis to the palmar articular surface of P3.
Distal Phalanx Rotation Angle (P3rotang)	Use dynamic angle function.<br>Line 1<br>• Dorsal P3 cortex<br>Line 2<br>• Dorsal hoof wall
Dorsal Dermal Wall Thickness (DDWT)	Reference Points: as for dorsal hoof wall thickness measurements.<br>Draw a 90° angle at each measurement location.<br>Measure distance from the dorsal cortex of P3 to the junction with the epidermis.
Dorsal Epidermal Wall Thickness (DEWT)	Reference Points: as for dorsal hoof wall thickness measurements.<br>Draw a 90° angle at each measurement location.<br>Measure distance from hoof wall margin to dermal-epidermal junction.
Dorsal Hoof Wall Thickness (DHWT)	Reference Points:<br>• Proximal: most proximal portion of dorsal P3 before it curves proximally to the extensor process.<br>• Distal: dorsodistal tip of P3<br>• Mid: halfway between the above measurements.<br>Draw a 90° angle at this location.<br>Measure distance from P3 cortex to the dorsal hoof wall margin.<br>If the radiographic marker is separate from the hoof wall, despite windowing, measure to the visible hoof wall.
Lateral Dermal Wall Thickness (LDWT) Medial Dermal Wall Thickness (MDWT)	Reference Points: as for lateral and medial hoof wall thickness<br>Measure horizontal distance between P3 and dermal/epidermal junction.
Lateral Epidermal Wall Thickness (LEWT) Medial Epidermal Wall Thickness (MEWT),	Reference points: as for lateral and medial hoof wall thickness<br>Measure horizontal distance between hoof wall and dermal/epidermal junction.
Lateral hoof wall thickness (LHWT), Medial hoof wall thickness (MHWT)	Reference points:<br>• Proximal – proximolateral and proximomedial P3<br>• Distal – distolateral and distomedial P3<br>• Lateral/medial hoof wall (on MRI, inside the bandage)<br>Measure horizontal distance between reference points.<br>On radiographs if marker appears not to touch hoof wall at location of measurement despite windowing, measure to visible hoof wall.

Proximal Middle Phalanx Angle (PrP2ang)	Use dynamic angle function:<br>Line 1<br>• Joint proximolateral and proximomedial P2<br>Line 2<br>• Radiographs: foot block<br>• MRI: horizontal guideline (plain images) OR line of best fit following solar surface of bandage (with WSHB)
Solar Angle of the Distal Phalanx (Sang)	Use dynamic angle function.<br>Line 1<br>• Radiographs: palmar P3<br>• MRI: most abaxial points of P3 in which the palmar process is still clearly visible.<br>Line 2<br>• Radiographs: horizontal along foot block<br>• MRI: horizontal guideline (plain images) OR line of best fit along solar surface of bandage (with the water-soaked hoof bandage (WSHB))<br>On radiographs, if there is a proximodistal imbalance in P3, place the first line halfway between the palmar processes.<br>For MRI, take lateral and medial measurements, then a mean gives the final SA value.
Sole Thickness	Reference point:<br>• Dorsodistal tip of P3<br>AND<br>• Radiographs: barium sulphate line on sole<br>• MRI: solar surface of bandage<br>Draw vertical line from dorsodistal tip of P3 to the solar reference point.
Toe Length	Reference points:<br>• Dorsodistal tip of P3<br>• Dorsodistal margin of hoof capsule (on MRI, inside bandage)<br>Measure distance between the reference points.
